# Saline versus 5% dextrose in water as a drug diluent for critically ill patients: a retrospective cohort study

**DOI:** 10.1186/s40560-020-00489-6

**Published:** 2020-09-11

**Authors:** Yukari Aoyagi, Takuo Yoshida, Shigehiko Uchino, Masanori Takinami, Shoichi Uezono

**Affiliations:** Intensive Care Unit, Department of Anesthesiology, The Jikei University Hospital, 3-19-18, Nishi-Shinbashi Minato-ku, Tokyo, 105-8471 Japan

**Keywords:** Critical care, Diluent, Saline, Dextrose in water, Hyperglycemia, Hypernatremia

## Abstract

**Background:**

The choice of intravenous infusion products for critically ill patients has been studied extensively because it can affect prognosis. However, there has been little research on drug diluents in this context. The purpose of this study is to evaluate the impact of diluent choice (saline or 5% dextrose in water [D5W]) on electrolyte abnormalities, blood glucose control, incidence of acute kidney injury (AKI), and mortality.

**Methods:**

This before-after, two-group comparative, retrospective study enrolled adult patients who stayed for more than 48 h in a general intensive care unit from July 2015 to December 2018. We changed the default diluent for intermittent drug sets in our electronic ordering system from D5W to saline at the end of 2016.

**Results:**

We included 844 patients: 365 in the D5W period and 479 in the saline period. Drug diluents accounted for 21.4% of the total infusion volume. The incidences of hypernatremia and hyperchloremia were significantly greater in the saline group compared to the D5W group (hypernatremia 27.3% vs. 14.6%, *p* < 0.001; hyperchloremia 36.9 % vs. 20.4%, *p* < 0.001). Multivariate analyses confirmed the similar effects (hypernatremia adjusted odds ratio (OR), 2.43; 95% confidence interval (CI), 1.54–3.82; hyperchloremia adjusted OR, 2.09; 95% CI, 1.31–3.34). There was no significant difference in the incidences of hyperglycemia, AKI, and mortality between the two groups.

**Conclusions:**

Changing the diluent default from D5W to saline had no effect on blood glucose control and increased the incidences of hypernatremia and hyperchloremia.

## Introduction

Management of serum electrolyte and glucose levels among critically ill patients is essential because these abnormalities have been reported to be associated with acute kidney injury (AKI) and mortality [1–4]. Thus, clinicians pay attention to the choice of intravenous infusion products which may have affected the abnormalities [5].

On the other hand, research focusing on the choice of drug diluents has been limited. We believe that the drug diluents may play an important role because critically ill patients generally require many types of drugs, including antibiotics and sedatives [6, 7]. The total amount of diluents administered may be high enough to introduce abnormalities in serum electrolyte and glucose levels. It is therefore necessary to investigate the impact of diluents on those abnormalities.

The purpose of the present study is to evaluate the effect of drug diluents in critically ill patients. We hypothesized that changing the default diluent from D5W to saline would improve blood glucose control without inducing electrolyte abnormalities.

## Materials and methods

We conducted a before-after, two-group comparative, observational study to retrospectively examine the effect of changing the drug diluent from D5W to saline. The study protocol was approved by The Jikei University Institutional Review Board (31-011[9510]). Because of the retrospective, observational nature of the study, the Board waived the need for written informed consent.

### Study setting and participants

We conducted this study in a mixed medical and surgical intensive care unit (ICU) with 20 beds at The Jikei University Hospital in Tokyo, Japan. During the period July 1, 2015, to December 31, 2018, consecutive patients admitted to the ICU were screened for inclusion. We included adult patients (age ≥ 20 years) who stayed in the ICU for more than 48 h. We excluded patients with ICU readmission during the same hospitalization period, those with no arterial line, those with end-stage kidney disease as defined by the Kidney Disease: Improving Global Outcomes classification [8], and those with a history of urinary diversion. All study patients were followed up until hospital discharge. We compared blood glucose control and electrolyte abnormalities during the D5W period (July 2015–December 2016) and the saline period (January 2017–December 2018).

### Change of our policy

Before the end of 2016, D5W was automatically selected if the doctor did not specify the diluent when ordering intermittent drugs by the computer ordering system. We observed unstable blood glucose levels in some patients, presumably owing to intermittent D5W infusion. Therefore, on January 1, 2017, we changed the default diluent for intermittent drug sets in our electronic ordering system from D5W to saline. The clinicians were allowed to change the diluent to D5W if deemed clinically necessary.

### Variables and outcomes

We assessed the following variables for 1 week or during the entire ICU stay, whichever was shorter: age, sex, height, weight, body mass index, comorbidities, time from hospitalization to ICU admission, patient category at ICU admission (planned surgical, nonplanned surgical, medical), main damaged organ, Acute Physiology and Chronic Health Evaluation (APACHE) III score [9], serum concentrations during the observation period (sodium, chloride, creatinine, blood glucose), type and volume of administered fluid, length of ICU and hospital stay, and ICU and hospital mortality. Serum concentrations of sodium and chloride were assessed by central laboratory analysis using a LABOSPECT 008α analyzer (Hitachi High-Technologies Corp, Tokyo, Japan), and blood glucose was assessed by blood gas analysis using an ABL800 FLEX analyzer (Radiometer Medical ApS, Copenhagen, Denmark). Samples were collected at 7:00 AM every morning. Blood gas samples were also collected at the discretion of the attending physician.

The primary outcomes were the incidences of hyperglycemia and hypernatremia during the observation period in consideration of the effect of D5W on blood glucose levels and the effect of saline on serum sodium levels, which may affect the clinical course [6]. The secondary outcomes were the incidences of hyperchloremia, hyponatremia, hypoglycemia, and AKI; blood glucose SD, AKI maximum stage, renal replacement therapy (RRT) requirement, ICU/hospital length of stay; and ICU/hospital mortality.

Hyperglycemia was defined as ≥ 180 mg/dL [6, 10–12]. Hypoglycemia was defined as < 70 mg/dL [13]. Hypernatremia was defined as ≥ 145 mmol/L [2, 5, 6, 14]. Hyponatremia was defined as < 135 mmol/L [2, 5, 6]. Hyperchloremia was defined as ≥ 110 mmol/L [5, 6, 15]. With respect to sodium, chloride, and blood glucose, the values at the time of ICU admission were defined as the baseline. With respect to creatinine, we defined the baseline value as the mean serum creatinine level measured 7 to 365 days before hospital admission [16]. If baseline creatinine data were not available, we estimated the level according to the equation for the Modification of Diet in Renal Disease for Japanese [17]. Diagnosis of AKI was made according to the Acute Kidney Injury Work Group’s Kidney Disease: Improving Global Outcomes definition [8].

### Statistical methods

Blood glucose standard deviation (SD) was calculated from all values measured during the observation period. Data are presented as median (interquartile range) or number (%), as appropriate. Univariable analyses for categoric variables were conducted using the chi-square or Fisher exact test. For continuous variables, comparisons were performed using the Mann-Whitney *U* test. For each outcome, patients with an abnormal baseline value were excluded from these analyses. The numbers of excluded patients were reported. To assess the effect of changing the diluent solution, we conducted multivariable analyses for hyperglycemia, hypernatremia, hyperchloremia, blood glucose SD, and AKI diagnosis. Variables in each model were selected according to clinical plausibility and previous studies [1–7, 14, 15, 18]. Multivariable logistic regression analysis was used to assess categoric outcomes, and linear regression analysis was used to assess continuous outcomes. Two-sided *p* values of < 0.05 were considered statistically significant. Analyses were performed using SPSS version 25.0 (IBM Corp, Armonk, New York, USA), Stata version 16 (StataCorp, College Station, Texas, USA), and R version 3.6.1 (The R Foundation for Statistical Computing, package Rcmdr/EZR) [19].

### Sensitivity analysis

For the assessment of the fluctuation of results due to different cutoff values for hypernatremia, hyperglycemia, and hyperchloremia, we performed logistic regression analyses with different cutoff values (Na 150 mmol/L; Cl 115 mmol/L; Glu 200 mg/dL) and linear regression analyses for the each highest value during the observation period.

## Results

**Fig. 1:**
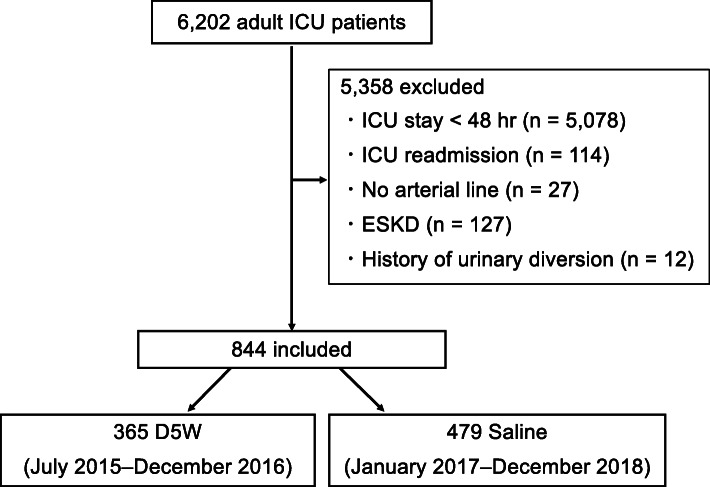
Patient flow. D5W, dextrose 5% in water; ESKD, end-stage kidney disease; ICU, intensive care unit

A total of 6202 adult patients were admitted to the ICU during the study period. Among those, 844 patients were included in the study: 365 and 479 in the D5W and saline period, respectively (Fig. 1).

Patient characteristics are listed in Table 1. The median age was 69 years, and 69.2% were men. The most commonly damaged organ was the cardiovascular system (40.5%), followed by the neurologic system (18.1%). The median levels of serum sodium, chloride, baseline creatinine, and blood glucose at ICU admission were all in the normal range. Patients in the saline group had fewer days from hospitalization to ICU admission and fewer planned surgical admissions than those in the D5W group. Otherwise, there was no significant difference between the two groups regarding patient characteristics.

**Table 1:** Patient characteristics

	Total (*N* = 844)	D5W (*n* = 365)	Saline (*n* = 479)	*p* value
Age, years	69 (55–77)	68 (56–75)	69 (55–78)	0.313
Male sex	584 (69.2)	244 (66.8)	340 (71.0)	0.202
Height, cm	164 (157–170)	164 (158–170)	164 (157–170)	0.593
Weight, kg	60 (50–70)	61 (51–70)	60 (50–70)	0.62
Body mass index	22 (20–25)	23 (20–25)	22 (20–25)	0.295
Comorbidity				
Heart failure	8 (0.9)	2 (0.5)	6 (1.3)	0.477
Respiratory failure	17 (2.0)	5 (1.4)	12 (2.5)	0.325
Liver cirrhosis	30 (3.6)	16 (4.4)	14 (2.9)	0.266
Hematologic malignancy	45 (5.3)	20 (5.5)	25 (5.2)	0.878
Metastatic malignancy	34 (4.0)	12 (3.3)	22 (4.6)	0.381
Immunosuppression	95 (11.3)	39 (10.7)	56 (11.7)	0.662
Time from hospitalization to ICU, days	2 (0–7)	3 (0–8)	1 (0–6)	0.005
Patient category				0.005
Planned surgical	291 (34.5)	150 (41.1)	141 (29.4)	
Nonplanned surgical	212 (25.1)	83 (22.7)	129 (26.9)	
Medical from ward	185 (21.9)	75 (20.5)	110 (23.0)	
Medical from ED	156 (18.5)	57 (15.6)	99 (20.7)	
Main damaged organ				0.745
Cardiovascular	342 (40.5)	156 (42.7)	186 (38.8)	
Neurologic	153 (18.1)	65 (17.8)	88 (18.4)	
Respiratory	127 (15.0)	49 (13.4)	78 (16.3)	
Digestive	120 (14.2)	51 (14.0)	69 (14.4)	
Other	102 (12.1)	44 (12.1)	58 (12.1)	
APACHE III score	71 (56–88)	71 (57–86)	72 (54–88)	0.916
Data at ICU admission				
Sodium, mmol/L	140 (137–142)	140 (137–142)	140 (137–143)	0.274
Chloride, mmol/L	107 (103–111)	108 (104–111)	107 (103–111)	0.105
Blood glucose, mg/dL	137 (115–169)	136 (113–169)	138 (115–169)	0.525
Baseline creatinine, mg/dL	0.85 (0.74–0.99)	0.84 (0.72–0.98)	0.85 (0.75–1.00)	0.448

Data are presented as median (interquartile range) or number (%)

*APACHE III* Acute Physiology and Chronic Health Evaluation III, *D5W* dextrose 5% in water, *ED* emergency department, *ICU* intensive care unit

Details of infusion volume during the observation period are shown in Table 2. Overall, 4845 mL was administered over a period of 3.99 days. Patients in the saline group received smaller volumes of D5W and larger volumes of saline than those in the D5W group. There was no significant difference between the two groups in the volume of other crystalloids and total volume of those infusions. Intermittent infusion considered as a drug diluent became 21.4% of the total infusion volume.

**Table 2:** Infusion volume, electrolyte and glucose abnormalities, acute kidney injury, and other outcomes

	Total (***N*** = 844)	D5W (***n*** = 365)	Saline (***n*** = 479)	***p*** value
Observation period, days	3.99 (2.87–6.80)	3.89 (2.88–6.44)	4.13 (2.86–7.00)	0.233
Continuous infusion				
Saline, mL	4 (0–145)	0 (0–126)	27 (0–164)	0.009
D5W, mL	254 (50–608)	257 (68–557)	253 (42–634)	0.851
Other crystalloids, mL	2492 (1506–3586)	2525 (1658–3620)	2430 (1359–3568)	0.427
Intermittent infusion				
Saline, mL	650 (150–1326)	150 (0–450)	1000 (650–1750)	< 0.001
D5W, mL	387 (0–916)	800 (535–1365)	0 (0–250)	< 0.001
Other crystalloids, mL	0 (0–500)	0 (0–500)	0 (0–500)	0.336
Total infusion, mL	4845 (3443–6,762)	4697 (3475–6502)	4883 (3381–6988)	0.541
Saline, mL	795 (250–1759)	207 (50–700)	1150 (700–2050)	< 0.001
D5W, mL	770 (312–1493)	1220 (752–1896)	418 (170–1003)	< 0.001
Other crystalloids, mL	2802 (1702–3988)	2850 (1894–3896)	2759 (1513–4036)	0.423
Hyperglycemia^*a*^	374/655 (57.1)	161/285 (56.5)	213/370 (57.6)	0.811
Hypernatremia^*b*^	160/735 (21.8)	47/321 (14.6)	113/414 (27.3)	< 0.001
Blood glucose SD, mg/dL^*c*^	22 (17–30)	21 (16–29)	23 (17–30)	0.235
Hypoglycemia^*d*^	88/833 (10.6)	29/359 (8.1)	59/474 (12.0)	0.053
Hyponatremia^*e*^	95/723 (13.1)	44/313 (14.1)	51/410 (12.4)	0.579
Hyperchloremia^*f*^	160/533 (30.0)	45/221 (20.4)	115/312 (36.9)	< 0.001
AKI diagnosis^*g*^	377/639 (59.0)	165/283 (58.3)	212/356 (59.5)	0.808
AKI maximum stage^*g*^				0.826
Stage 1	204 (54.1)	85 (51.5)	119 (56.1)	
Stage 2	132 (35.0)	61 (37.0)	71 (33.5)	
Stage 3	41 (10.9)	19 (11.6)	22 (10.4)	
RRT requirement^*g*^	20 (3.1)	6 (2.1)	14 (3.9)	0.254
ICU LOS, days	4.0 (2.9–6.8)	3.9 (2.9–6.4)	4.1 (2.9–7.3)	0.269
Hospital LOS, days	38 (23–62)	40 (24–62)	37 (23–62)	0.497
ICU mortality	50 (5.9)	24 (6.6)	26 (5.4)	0.557
Hospital mortality	125 (14.8)	56 (15.3)	69 (14.4)	0.769

Data are presented as median (interquartile range) or number (%)

*AKI* acute kidney injury, *D5W* dextrose 5% in water, *ICU* intensive care unit, *LOS* length of stay, *RRT* renal replacement therapy, *SD* standard deviation

^*a*^Patients with baseline blood glucose ≥ 180 mg/dL were excluded (189 patients)

^*b*^Patients with baseline sodium ≥ 145 mmol/L were excluded (109 patients)

^*c*^Patients with blood glucose < 70 mg/dL or ≥ 180 mg/dL were excluded (200 patients)

^*d*^Patients with baseline blood glucose < 70 mg/dL were excluded (11 patients)

^*e*^Patients with baseline sodium < 135 mmol/L were excluded (121 patients)

^*f*^Patients with baseline chloride ≥ 110 mmol/L were excluded (311 patients)

^*g*^Patients who received a diagnosis of AKI at ICU admission were excluded (205 patients)

Unadjusted primary and secondary outcomes are shown in Table 2. There was no significant difference in the incidence of hyperglycemia between the two groups. The incidence of hypernatremia was significantly greater in the saline group than in the D5W group. Of the secondary outcomes, only the incidence of hyperchloremia was different, being greater in the saline group than in the D5W group.

**Fig. 2:**
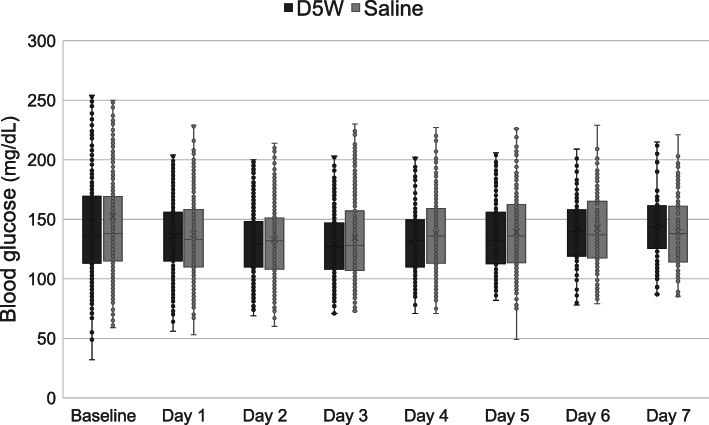
Box plots for daily blood glucose levels. The box plots show median and interquartile range. D5W, dextrose 5% in water

**Fig. 3:**
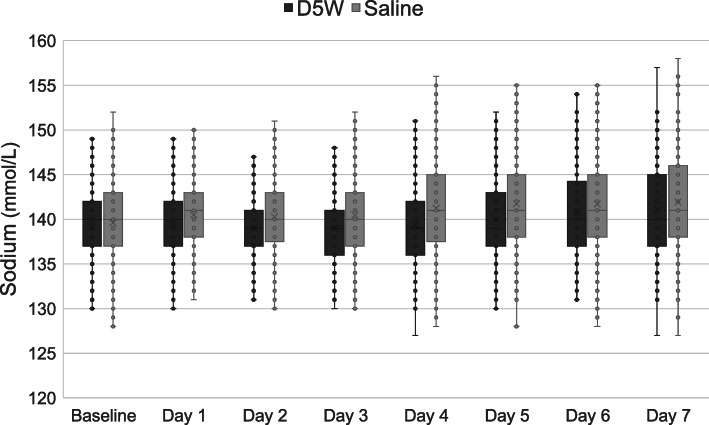
Box plots for daily serum sodium levels. The box plots show median and interquartile range. D5W, dextrose 5% in water

**Fig. 4:**
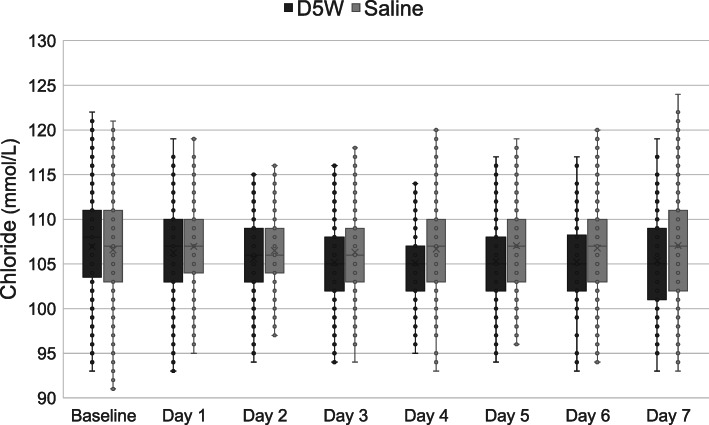
Box plots for daily serum chloride levels. The box plots show median and interquartile range. D5W, dextrose 5% in water

Box plots for daily serum blood glucose, sodium, and chloride levels are shown in Figs. 2, 3 and 4, respectively. Patients in the saline group had greater sodium levels on days 1 to 5 and greater chloride levels on days 2 to 7.

Results of multivariable regression analyses for primary outcomes are shown in Table 3 (for secondary outcomes are shown in Table 4). In the adjusted models, the diluent group was independently associated with hypernatremia (adjusted odds ratio (OR), 2.43; 95% confidence interval (CI), 1.54–3.82) and hyperchloremia (adjusted OR, 2.09; 95% CI, 1.31–3.34). There was no significant association between the diluent group and hyperglycemia (adjusted OR, 1.08; 95% CI, 0.77–1.5), blood glucose SD (beta coefficient, 0.4; 95% CI, − 1.37 to 2.18), or AKI (adjusted OR, 1.11; 95% CI, 0.79–1.56) in the adjusted model. The results of sensitivity analyses are shown in supplemental Table 1 and 2. Results of the analyses were similar to those in the main ones.

**Table 3:** Multivariable regression analyses for primary outcomes

Outcome	Adjusted OR/β-coefficient (95% CI)	*p* value
Hyperglycemia^*a*^		
Diluent group (saline)	**1.08 (0.77–1.5)**	**0.671**
Blood glucose at ICU admission, mg/dL	1.01 (1.01–1.02)	< 0.001
Age, year	1.01 (1.0–1.02)	0.097
Weight, kg	1.0 (0.99–1.01)	0.926
APACHE III score	1.02 (1.01–1.03)	< 0.001
Time from hospitalization to ICU, days	1.01 (0.99–1.02)	0.339
Patient category		
Planned surgical	Reference	
Nonplanned surgical	0.73 (0.48–1.13)	0.158
Medical from ward	1.11 (0.65–1.91)	0.700
Medical from ED	0.87 (0.54–1.42)	0.582
Hypernatremia^*b*^		
Diluent group (Saline)	**2.43 (1.54–3.82)**	**< 0.001**
Sodium at ICU admission, mmol/L	1.18 (1.11–1.26)	< 0.001
Age, year	1.01 (0.99–1.02)	0.373
Weight, kg	1.0 (0.98–1.02)	0.997
APACHE III score	1.02 (1.01–1.03)	< 0.001
Time from hospitalization to ICU, days	1.01 (1.0–1.02)	0.070
Patient category		
Planned surgical	Reference	
Nonplanned surgical	1.74 (0.97–3.12)	0.061
Medical from ward	2.53 (1.33–4.82)	0.005
Medical from ED	1.70 (0.89–3.27)	0.109

*APACHE III* Acute Physiology and Chronic Health Evaluation III, *ED* emergency department, *ICU* intensive care unit, *OR* odds ratio

^*a*^Patients with baseline blood glucose ≥ 180 mg/dL were excluded (189 patients)

^*b*^Patients with baseline sodium ≥ 145 mmol/L were excluded (109 patients)

**Table 4:** Multivariable regression analyses for secondary outcomes

Outcome	Adjusted OR/β-coefficient (95% CI)	*p* value
Hyperchloremia^*a*^		
Diluent group (Saline)	**2.09 (1.31–3.34)**	**0.002**
Chloride at ICU admission, mmol/L	1.15 (1.08–1.22)	< 0.001
Age, year	1.0 (0.98–1.02)	0.976
Weight, kg	1.0 (0.99–1.02)	0.632
APACHE III score	1.02 (1.01–1.03)	< 0.001
Time from hospitalization to ICU, days	1.0 (0.99–1.01)	0.88
Patient category		
Planned surgical	Reference	
Unplanned surgical	2.37 (1.16–4.83)	0.018
Medical from ward	2.23 (1.02–4.88)	0.044
Medical from ED	1.88 (0.86–4.09)	0.112
Blood glucose SD^*b*^		
Diluent group (Saline)	**0.4 (− 1.37 to 2.18)**	**0.655**
Blood glucose at ICU admission, mg/dL	0.03 (0–0.07)	0.089
Age, year	0.08 (0.02–0.15)	0.01
Weight, kg	**−** 0.07 (**−** 0.14 to **−** 0.01)	0.029
APACHE III score	0.06 (0.02–0.98)	0.006
Time from hospitalization to ICU, days	0.02 (**−** 0.04 to 0.09)	0.490
Patient category		
Planned surgical	Reference	
Unplanned surgical	0.31 (**−** 1.96 to 2.58)	0.788
Medical from ward	5.72 (2.91–8.52)	< 0.001
Medical from ED	0.87 (**−** 1.71 to 3.44)	0.51
AKI diagnosis^*c*^		
Diluent group (Saline)	**1.11 (0.79–1.56)**	**0.542**
Creatinine at ICU admission, mg/dL	2.57 (1.31–5.05)	0.006
Chloride at ICU admission, mmol/L	1.0 (0.96–1.03)	0.816
Age, year	0.99 (0.98–1.01)	0.347
Weight, kg	1.01 (0.99–1.02)	0.273
APACHE III score	1.02 (1.01–1.03)	< 0.001
Time from hospitalization to ICU, days	0.99 (0.98–1.0)	0.034
Patient category		
Planned surgical	Reference	
Unplanned surgical	0.43 (0.27–0.68)	< 0.001
Medical from ward	0.94 (0.51–1.75)	0.854
Medical from ED	1.31 (0.72–2.38)	0.372

^*a*^Patients with baseline chloride ≥ 110 mmol/L were excluded (311 patients)

^*b*^Patients with blood glucose < 70 mg/dL or ≥ 180 mg/dL were excluded (200 patients)

^*c*^Patients who received a diagnosis of AKI at ICU admission were excluded (205 patients)

*AKI* acute kidney injury, *APACHE III* Acute Physiology and Chronic Health Evaluation III, *ED* emergency department, *ICU* intensive care unit, *OR* odds ratio, *SD* standard deviation

## Discussion

### Key findings

We conducted a before-after, two-group comparative study that examined the effect of changing the drug diluent from D5W to saline. Patients in the saline group received smaller volumes of D5W and larger volumes of saline compared with the D5W diluent group. Contrary to our initial expectation, there was no significant difference in the incidence of hyperglycemia or blood glucose SD. However, the incidence of hypernatremia was significantly greater in the saline group than in the D5W group. There was no significant difference in incidence of AKI or mortality between the two groups.

### Comparison with previous studies

There are few previous studies that evaluated the effect of diluent on patient outcomes [6, 7]. One before-after study, in which the drug diluent was changed from saline to D5W, reported that the incidence of hyperchloremia decreased (adjusted OR, 0.50; 95% CI, 0.27–0.94), without difficulty in blood glucose control [6]. That study reported that diluents accounted for a large part of the total infusion volume (63%). In contrast, the diluent amounted to just over 20% of all of the administered formulations in our present study. However, even with such a small percentage, changing diluent from D5W to saline increased the incidences of hypernatremia and hyperchloremia. These results highlight the magnitude of the effect of diluent choice on electrolyte abnormalities, consistent with the previous studies [6, 7].

Regarding the incidence of hyperglycemia, the difference between the D5W group and saline group was not reported in the previous study (82.3% vs. 88.1%) [6]. Our present study found that changing default diluents from D5W to saline did not improve the incidence of hyperglycemia, which is consistent with the previous study [6]. We also found no significant difference in blood glucose SD between the two groups, implying glucose stability. This might be because the amount of glucose in D5W (2.5 g in 50 mL or 5 g in 100 mL) is too small to affect blood glucose values. In addition, blood glucose has an adjustment medicine, insulin, as an antagonist, unlike electrolytes.

Hypernatremia is thought to be due to unreplaced water loss, water loss into cells, or excess sodium. Among those mechanics, hypernatremia induced by infusion may be based on sodium overload, which is regarded as iatrogenic hypernatremia. Hypernatremia has been reported to be associated with increased mortality. In a secondary analysis of the European Surgical Outcome Study, which provided data describing 46,539 patients undergoing inpatient noncardiac surgery, moderate to severe hypernatremia was independently associated with mortality (adjusted OR, 3.4; 95% CI, 2.0–6.0) [3]. Similarly, a relation between hyperchloremia and AKI/mortality has been suggested [20, 21]. A systematic review and meta-analysis to assess the relation between the chloride content of intravenous resuscitation fluids and patient outcomes for the perioperative or intensive care setting showed that high-chloride fluids were associated with the incidence of AKI [20]. However, in the present study, there was no significant difference in AKI incidence or mortality between the two groups, possibly owing to lack of adequate sample size to evaluate these outcomes.

### Significance and implications

We focused on the effect of the choice of drug diluent on outcomes in critically ill patients. We changed the diluent from D5W to saline for the purpose of improving blood glucose control based on our experience with several patients. However, we found no effect on glucose control and did find significant effects on the incidences of hypernatremia and hyperchloremia. Considering the results of the present study in conjunction with previous studies, D5W appears to be a better choice as default diluent for critically ill patients, although it is necessary to select diluents according to the condition of individual patients. Since only observational studies, including the present study, have been conducted on this issue, randomized interventional studies are needed to confirm the findings.

### Strengths and limitations

We believe that the present study has the largest sample size among the studies assessing the choice of drug diluents for critically ill patients [6, 7]. Furthermore, we used the Kidney Disease: Improving Global Outcomes classification [8] to diagnose AKI, which includes more detailed diagnostic criteria compared with those used in the previous studies. However, our study has several limitations. First, there were confounders that could not be included due to the retrospective observational nature of our study, e.g., past history of diabetes and physiological status. Second, because this was a single-center study and the results might have been influenced by the local clinical practice, our findings may limit the generalizability. Third, we excluded patients without arterial line, those with end-stage kidney disease, and those with abnormal baseline values of serum electrolytes and glucose, possibly leading to selection bias. Fourth, owing to the before-after study design, there was a difference in the duration of observation between the two groups (D5W 18 months; saline 24 months). This is because we changed the protocol for blood glucose control in July 2015. In addition, there were some differences in the patient background (days from hospitalization to ICU admission and scheduled surgery). However, our findings in the multivariate analyses including those factors found similar results to those in the univariate analyses. Fifth, some drugs require to be dissolved in saline or D5W only, which cannot be changed by physicians. We could not evaluate the impact of these drugs. Finally, we did not collect information for adverse events related to electrolyte abnormalities other than AKI and mortality. Therefore, we could not show the comprehensive impact for the choice of drug diluents. Further prospective studies are warranted.

## Conclusions

Changing the drug diluent default from D5W to saline had no effect on blood glucose control but increased the incidence of hypernatremia and hyperchloremia, without affecting the incidence of AKI or mortality. D5W may be a better choice as a default diluent for critically ill patients. Randomized interventional studies are warranted to confirm the findings.

## Supplementary information

**Additional file 1: Supplemental Table 1.** Sensitivity analyses for multivariable logistic regression. **Supplemental Table 2.** Sensitivity analyses for multivariable linear regression.

## Data Availability

The datasets used and/or analyzed during the current study are available from the corresponding author on reasonable request. For more information please email our Research Data Team.

## Abbreviations

D5W: 5% dextrose in water
AKI: Acute kidney injury
ICU: Intensive care unit
APACHE III: Acute Physiology and Chronic Health Evaluation III
SD: Standard deviation
RRT: Renal replacement therapy
OR: Odds ratio
CI: Confidence interval
