# Supplementary material for: Saline versus 5% dextrose in water as a drug diluent for critically ill patients: a retrospective cohort study
Source: J Intensive Care. 2020 Sep 11;8:69. doi: 10.1186/s40560-020-00489-6 (PMC7488509; doi:10.1186/s40560-020-00489-6)
Supplement: Supplementary file 1 — Additional file 1: Supplemental Table 1. Sensitivity analyses for multivariable logistic regression. Supplemental Table 2. Sensitivity analyses for multivariable linear regression. [file 40560_2020_489_MOESM1_ESM.docx]

**Supplemental Table 1. Sensitivity analyses for multivariable logistic regression**

| Outcome | Adjusted OR (95% CI) | *p* Value |
| --- | --- | --- |
| Hyperglycemia*^a^* |  |  |
| Diluent group (Saline) | **0.95 (0.69–1.32)** | **0.767** |
| Blood glucose at ICU admission, mg/dL | 1.01 (1.01–1.02) | < 0.001 |
| Age, yr | 1.01 (1.0–1.02) | 0.229 |
| Weight, kg | 1.01 (0.99–1.02) | 0.262 |
| APACHE III score | 1.02 (1.01–1.03) | < 0.001 |
| Time from hospitalization to ICU, d | 1.0 (0.99–1.01) | 0.516 |
| Patient category |  |  |
| Planned surgical | Reference |  |
| Nonplanned surgical | 0.80 (0.53–1.22) | 0.304 |
| Medical from ward | 1.57 (0.96–2.59) | 0.073 |
| Medical from ED | 0.84 (0.51–1.37) | 0.478 |
|  |  |  |
| Hypernatremia*^b^* |  |  |
| Diluent group (Saline) | **2.03 (1.07–3.88)** | **0.031** |
| Sodium at ICU admission, mmol/L | 1.14 (1.07–1.22) | < 0.001 |
| Age, yr | 1.03 (1.00–1.05) | 0.036 |
| Weight, kg | 1.00 (0.98–1.03) | 0.677 |
| APACHE III score | 1.02 (1.01–1.04) | < 0.001 |
| Time from hospitalization to ICU, d | 1.00 (0.99–1.01) | 0.689 |
| Patient category |  |  |
| Planned surgical | Reference |  |
| Nonplanned surgical | 2.71 (1.13–6.50) | 0.025 |
| Medical from ward | 4.16 (1.64–10.59) | 0.003 |
| Medical from ED | 2.42 (0.90–6.51) | 0.079 |
|  |  |  |
| Hyperchloremia^c^ |  |  |
| Diluent group (Saline) | **2.12 (1.17–3.86)** | **0.014** |
| Chloride at ICU admission, mmol/L | 1.12 (1.05–1.19) | 0.001 |
| Age, yr | 1.02 (1.0–1.05) | 0.075 |
| Weight, kg | 1.01 (1.0–1.03) | 0.299 |
| APACHE III score | 1.02 (1.01–1.03) | < 0.001 |
| Time from hospitalization to ICU, d | 1.0 (0.99–1.01) | 0.823 |
| Patient category |  |  |
| Planned surgical | Reference |  |
| Nonplanned surgical | 2.33 (1.07–5.06) | 0.033 |
| Medical from ward | 3.5 (1.42–8.6) | 0.006 |
| Medical from ED | 1.76 (0.65–4.74) | 0.265 |

*^a^*Patients with baseline blood glucose ≥ 200 mg/dL were excluded (121 patients).

*^b^*Patients with baseline sodium ≥ 150 mmol/L were excluded (14 patients).

*^c^*Patients with baseline chloride ≥ 115 mmol/L were excluded (68 patients).

OR = odds ratio, CI: confidence interval, ICU = intensive care unit, APACHE III = Acute Physiology and Chronic Health Evaluation III, ED = emergency department.

**Supplemental Table 2.** **Sensitivity analyses for multivariable linear regression**

| Outcome | β-Coefficient (95% CI) | *p* Value |
| --- | --- | --- |
| Highest blood glucose*^a^* |  |  |
| Diluent group (Saline) | **1.17 (-6.24 to 8.58)** | **0.756** |
| Blood glucose at ICU admission, mg/dL | 0.35 (0.21–0.49) | < 0.001 |
| Age, yr | 0.26 (-0.01 to 0.54) | 0.058 |
| Weight, kg | 0 (-0.27 to 0.28) | 0.975 |
| APACHE III score | 0.35 (0.18–0.51) | < 0.001 |
| Time from hospitalization to ICU, d | 0.11 (-0.15 to 0.37) | 0.408 |
| Patient category |  |  |
| Planned surgical | Reference |  |
| Nonplanned surgical | -3.89 (-13.45 to 5.67) | 0.424 |
| Medical from ward | 14.21 (2.56–25.86) | 0.017 |
| Medical from ED | -3.94 (-14.77 to 6.88) | 0.475 |
|  |  |  |
| Highest blood sodium*^b^* |  |  |
| Diluent group (Saline) | **1.58 (0.61–2.56)** | **0.002** |
| Sodium at ICU admission, mmol/L | 0.4 (0.27–0.52) | < 0.001 |
| Age, yr | 0.01 (-0.26 to 0.47) | 0.583 |
| Weight, kg | 0.01 (-0.29 to 0.40) | 0.757 |
| APACHE III score | 0.03 (0.01–0.05) | 0.008 |
| Time from hospitalization to ICU, d | 0.02 (-0.01 to 0.04) | 0.200 |
| Patient category |  |  |
| Planned surgical | Reference |  |
| Nonplanned surgical | 0.52 (-0.80 to 1.85) | 0.438 |
| Medical from ward | 0.48 (-1.09 to 2.05) | 0.546 |
| Medical from ED | 0.46 (-1.01 to 1.93) | 0.537 |
|  |  |  |
| Highest blood chloride^c^ |  |  |
| Diluent group (Saline) | **1.83 (0.69–2.96)** | **0.002** |
| Chloride at ICU admission, mmol/L | 0.39 (0.27–0.51) | < 0.001 |
| Age, yr | 0.01 (-0.03 to 0.05) | 0.604 |
| Weight, kg | 0.01 (-0.02 to 0.05) | 0.457 |
| APACHE III score | 0.03 (0.01–0.05) | 0.011 |
| Time from hospitalization to ICU, d | 0.02 (-0.01 to 0.04) | 0.179 |
| Patient category |  |  |
| Planned surgical | Reference |  |
| Nonplanned surgical | 1.30 (-0.45 to 3.06) | 0.145 |
| Medical from ward | 0.19 (-1.73 to 2.11) | 0.848 |
| Medical from ED | 0.91 (-0.93 to 2.74) | 0.333 |

*^a^*Patients with blood glucose < 70 mg/dL or ≥ 180 mg/dL were excluded (200 patients).

*^b^*Patients with baseline sodium ≥ 145 mmol/L were excluded (109 patients).

*^c^*Patients with baseline chloride ≥ 110 mmol/L were excluded (311 patients).

CI: confidence interval, APACHE III = Acute Physiology and Chronic Health Evaluation III, ICU = intensive care unit, ED = emergency department.
